# Anticorrosion Coated Stainless Steel as Durable Support for C-N-TiO_2_ Photo Catalyst Layer

**DOI:** 10.3390/ma13194426

**Published:** 2020-10-05

**Authors:** Emile Salomon Massima Mouele, Mihaela Dinu, Anca Constantina Parau, Alina Vladescu, Myo Tay Zar Myint, Htet Htet Kyaw, Jamal Al-Sabahi, Mohammed Al-Abri, Sergey Dobretsov, Mohammed A. Al Belushi, Rahma Al-Mamari, Mariana Braic, Leslie Felicia Petrik

**Affiliations:** 1 Environmental and Nano Sciences Group, Department of Chemistry, University of the Western Cape, Bellville 7535, South Africa; 2 Research Center for Advanced Surface Processing and Analysis by Vacuum Technologies (ReCAST), National Institute for Optoelectronics (INOE) 2000, 409 Atomistilor St., Magurele, 077125 Bucharest, Romania; mihaela.dinu@inoe.ro (M.D.); anca.parau@inoe.ro (A.C.P.); alinava@inoe.ro (A.V.); mariana.braic@inoe.ro (M.B.); 3 Research Center for Physical Materials Science and Composite Materials, National Research Tomsk Polytechnic University, Lenin Avenue 43, Tomsk 634050, Russia; 4 Department of Physics, College of Science, Sultan Qaboos University, P. O. Box 36, 123 Al-Khoud, Muscat 123, Oman; myomyint@squ.edu.om; 5 Nanotechnology Research Center, Sultan Qaboos University, P. O. Box 33, Al-Khoud, Muscat 123, Oman; htethtetkyaw2006@gmail.com (H.H.K.); jamal@squ.edu.om (J.A.-S.); alabri@squ.edu.om (M.A.-A.); 6 Petroleum and Chemical Engineering Department, Sultan Qaboos University, P. O. Box 33, Al-Khoud, Muscat 123, Oman; 7 Department of Marine Science and Fisheries, Sultan Qaboos University, P. O. Box 34, Al-Khoud, Muscat 123, Oman; sergey@squ.edu.om (S.D.); hamoodbeluch@gmail.com (M.A.A.B.); rahmamari93@gmail.com (R.A.-M.); 8 Center of Excellence in Marine Biotechnology, Sultan Qaboos University, P. O. Box 50, Al-Khoud, Muscat 123, Oman

**Keywords:** wastewater, dye decolouration, pollutants, advanced oxidation processes, anticorrosion, coating, photo catalysis, antimicrobial activity

## Abstract

The development of durable photocatalytic supports resistant in harsh environment has become challenging in advanced oxidation processes (AOPs) focusing on water and wastewater remediation. In this study, stainless steel (SS), SS/Ti (N,O) and SS/Cr-N/Cr (N,O) anticorrosion layers on SS meshes were dip-coated with sol gel synthesised C-N-TiO_2_ photo catalysts pyrolysed at 350 °C for 105 min, using a heating rate of 50 °C/min under N_2_ gas. The supported C-N-TiO_2_ films were characterised by scanning electron microscopy (SEM) coupled with energy dispersive spectroscopy (EDS), X-ray diffraction (XRD) and Raman spectroscopy. The results showed that C-N-TiO_2_ was successfully deposited on anticorrosion coated SS supports and had different morphologies. The amorphous C and TiO_2_ were predominant in C-N-TiO_2_ over anatase and rutile phases on the surface of SS and anticorrosion supports. The C-N-TiO_2_ coated films showed enhanced photocatalytic activity for the decolouration of O.II dye under both solar and UV radiations. The fabricated C-N-TiO_2_ films showed significant antibacterial activities in the dark as well as in visible light. Herein, we demonstrate that SS/Ti(N,O) and SS/Cr-N/Cr(N,O) anticorrosion coatings are adequate photocatalytic and corrosion resistant supports. The C-N-TiO_2_ photo catalytic coatings can be used for water and wastewater decontamination of pollutants and microbes.

## 1. Introduction

The removal of recalcitrant organic pollutants such as dyes, pharmaceuticals, and personal care products in industrial wastewater effluents and sewage has been the subject of research in recent years [1,2,3,4,5,6,7]. On the other hand, the presence of micro-organisms in water and wastewater treatment effluents and distribution systems has resulted in a challenge to reclaim potable water from unconventional sources. The consumption of pathogen-contaminated water has exposed low-income populations to water-related sicknesses such as diarrhea, typhoid fever, cholera, giardia, dysentery, etc., which in return have devastated communities worldwide [8,9,10]. Various treatment methods such as adsorption on activated carbon, ozonation, reverse osmosis, ion exchange on synthetic adsorbent resins, flocculation, etc. have been developed [1,11,12,13].

However, most of these methods have high operating costs and/or are inefficient due to the complexity of the aromatic structures of persistent organic pollutants [13]. Likewise, the resistance of pathogens and micro-organisms in treatment systems often requires the use of excessive amounts of disinfectants such as chlorine, which in turn incur high costs for their removal [14]. The existing methods developed for bulk water sterilisation still show some limitations. Typically, exposure of bacterial colonies to toxic compounds such as ethylene oxide or chlorine gas followed by high temperature and pressure are common techniques for the deactivation of micro-organisms [15]. However, previous reports claimed that some of these procedures could damage/destroy the equipment used to kill micro-organisms and often suffer from reduced efficacies [16]. Therefore, there is a need for the development of new and appropriate advanced treatment protocols that are capable of not only degrading organic pollutants but also are efficient at eliminating micro-organisms from water sources.

Advanced oxidation processes (AOPs) are considered as robust techniques capable of degrading organic contaminants in water and wastewater, converting them into harmless substances without any post-treatment processes needed [17,18,19,20,21,22,23,24]. AOPs are based on the generation of the hydroxyl radical, a strong oxidation agent that can completely degrade organic contaminants into forms such as CO_2_, water and simple salts [4,20,22,24,25]. AOPs induced by photo catalysis under solar and UV light are promising techniques for the removal of POPs and deactivation of pathogenic micro-organisms from water sources [26,27]. When irradiated, photo catalysis using semi-conductor catalysts such as TiO_2_, ZnO, etc., produces diverse reactive oxygen species, including O_2_**^−^**, O**^.^**, H_2_O_2_ and mostly non-selective OH**^.^** that efficiently terminates POPs and micro-organisms [28]. In AOPs, heterogeneous photo catalysts such as TiO_2_ have been used to accelerate the production of free radicals by both oxidation and reduction processes [4]. TiO_2_ has been used as a convenient photo catalyst due to its low cost, high stability and exceptional photo catalytic effectiveness [29]. Consequently, various classes of water pollutants including azo dyes and microorganisms have been treated using TiO_2_-based photo catalysis [13,21,29,30,31].

Research also supports that semiconductor catalysts can be doped to control the band gap and reduce the electron-hole recombination rate, which in turn may improve the activity of the catalyst [32,33,34,35,36,37]. Moreover, literature also states that semiconductor photo catalysts in their single or co-doped form can be deposited on particular supports to overcome the post-separation dilemma experienced with powder catalysts [38,39]. Even though previous studies reported that the coating process may decrease the specific surface area of the catalysts [40,41], coating of various material supports such as SS has been conducted [42,43,44,45]. Apart from these, Zhang and Wang [46] reported that prolonged exposure of stainless steel (SS) in oxidizing or acidic environments may result in its corrosion mostly when the Cr_2_O_3_ passive layer is scratched and may cause metal rusting, loss of thickness and weight. This could lead to contamination of water effluents being treated. Hence, the corrosion of SS in the oxidizing environment needs to be overcome to achieve the desired removal of the pollutant and avoid undesired water toxicity. SS mesh (304 L grade) was coated with Ti and Cr transition metal-based nitrides and oxynitrides as both mono- and double-protective layers by cathodic arc evaporation (CAE) method [47,48]. The anticorrosion behaviour of the obtained SS/Ti(N,O) and SS/Cr-N/Cr(N,O) coatings was also investigated and were found to be corrosion resistant in acidic environments [49].

In this study, SS, SS/Ti (N,O) and SS/Cr-N/Cr (N,O) anticorrosion meshes were evaluated as supports for C-N-TiO_2_ nanocatalysts, so as to study their stability and photo catalytic performance to degrade pollutants (dyes) and microorganisms.

## 2. Materials and Methods 

The 304L steel was acquired from Bibus Metals SRL (subsidiary of Bibus Holding AG, Switzerland). According to the certificate of quality, the chemical composition in wt. % provided by the manufacturer was: 70.976% Fe, 0.004% C, 1.220% Mn, 0.208% Si, 17.746% Cr, 8.524% Ni, 0.020% P, 0.014% S, 0.160% Co, 0.589% Mo, and 0.539% Cu.

The following chemicals: polyacrylonitrile (PAN) powder (99.5%, Good fellow, Huntingdon, England), titanium tetrachloride (MW 189.68 g/mol) and titanium (IV) oxide (powder) Degussa (99.5%, Sigma Aldrich, Johannesburg, South Africa), N,N dimethyl formamide (DMF) (99%) and ammonium nitrate, ACS (95%), Industrial Analytical (Pty), Johannesburg, South Africa), sulfuric acid (98%) and sodium hydroxide flakes CP (97%), kimix, Cape Town, South Africa), and orange II sodium salt (85%, Sigma Aldrich, Johannesburg, South Africa) were subsequently used for the synthesis of carbon-nitrogen co-doped catalysts (C-N-TiO_2_) and photocatalytic decolouration of orange II dye.

**Figure 1:**
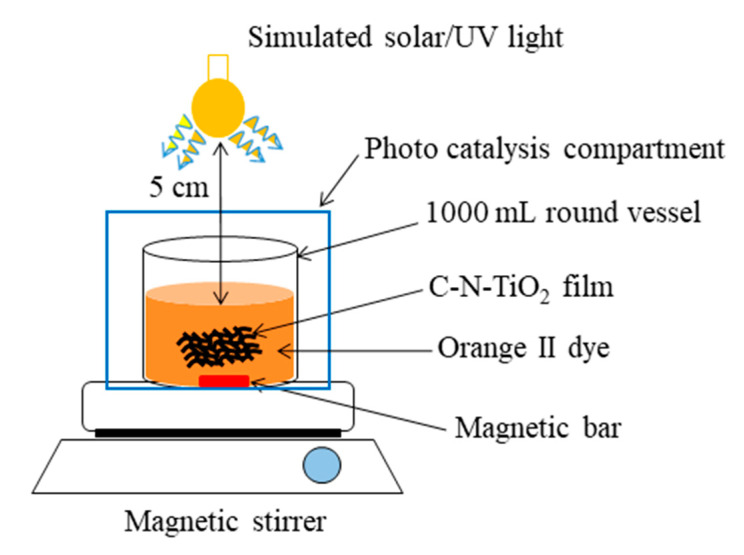
Photo catalysis set up for the degradation of orange II dye at the following fixed parameters: O.II concentration 10 mg/L, volume 500 mL, solution pH 2.5, A solar simulator light ((AM 1.5 radiation, 100 mW/cm^2^) and a UV lamp (Mega-Ray 160 W/240 V MR160 SPL11/14 from Kimix) and irradiation time 120 min. Varied parameters: type of uncoated or coated meshes.

The following materials: ceramic crucibles, magnetic stand, and a 3-zone horizontal ceramic-tube furnace (Brother XD 1600MT manufactured by Zhengzhou Brother Furnace Co, LNpt clear TD, Electronic Industrial Town Zhanggong Pu, Xiu wu county, Jiaozuo, China) were used to calcine the C-N-TiO_2_-coated meshes. The used light sources were simulated solar light (AM 1.5 radiation, 100 mW/cm^2^) obtained from a solar simulator (Sciencetech SS1.6 kW, London, ON, Canada) for photo degradation. LUX meter (ISO-TECH ISM 410) was used to determine the location that achieved the required light intensity, and a UV lamp (Mega-Ray 160 W/240 V MR160 SPL11/14 from Kimix, Cape Town, South Africa) was applied in a photo catalytic system (Figure 1) to perform photo catalysis experiments. The anticorrosion SS/Ti(N,O) and SS/Cr-N/Cr(N,O) coatings were designed and it was previously demonstrated to be resistant to corrosion in acidic environments according to Dinu et al. [49] and Pana et al. [50].

All meshes were tested in the same size of 2 cm wide by 6 cm long (2 cm × 6 cm and hole diameter: 1 mm). Marine Broth (Hi-media, India) and Marine agar (Hi-media, India) were used to prepare bacterial media for antimicrobial experiment with *Bacillus* *subtilis* (SQUMSF005). *Bacillus* *subtilis* is a marine biofouling bacteria and it was isolated from a reverse osmosis membrane of a desalination plant [51].

### 2.1. Experimental—Synthesis of C-N-TiO_2_ Sol Gel, Dip Coating of Stainless Steel and Anticorrosion Meshes and Calcination under N_2_ Gas

The Cr and Ti-based nitride and oxynitride (SS/Ti(N,O) and SS/Cr-N/Cr(N,O)) corrosion coatings were prepared by reactive cathodic arc evaporation (CAE) at the applied conditions according to Dinu et al. [49].

The C-N-TiO_2_ sol-gel was synthesised by the dissolution of 8 g PAN in 100 mL of 99% DMF followed by the addition of 3 mL TiCl_4_ and 3 mL 5% NH_4_NO_3_ in a 200 mL capped borosilicate glass bottle that was stirred for 24 h at room temperature [52]. Stainless steel (SS) meshes and their anticorrosion coated meshes were carefully cleaned with acetone, ethanol and water and dried in an oven at 60 °C for 30 min prior to remove impurities.

About 40 mg of the prepared C-N-TiO_2_ sol gel was loaded on the clean and dried stainless steel meshes by dip coating technique. The coated meshes were placed on clean and dried sample holders (crucibles), which were positioned at the centre of the heating zone. The samples were then calcined at a chosen temperature of 350 °C at a heating rate of 50 °C /min in a furnace for a holding time of 105 min under nitrogen gas at flow rate of 20 mL/min. The calcination temperature, ramping rate and holding time on the furnace were manually set following the instrument guidelines. The system was allowed to cool down under N_2_ flow until dark annealed C-N-TiO_2_ films were obtained. The SS mesh coated with anticorrosion SS/Ti (N,O) and SS/Cr-N/Cr (N,O) layers was dip coated with the C-N-TiO_2_ sol-gel in a similar manner and calcined following the same procedure.

### 2.2. Characterisation of C-N-TiO_2_ Nano Films

The elemental composition of uncoated SS and discs coated by anticorrosion SS/Ti (N,O) and SS/Cr-N/Cr (N,O) coatings were scrutinised by energy dispersive X-ray spectrometer (EDS) (Bruker, Billerica, MA, USA). To recall, the elemental composition of uncoated SS and anticorrosion SS/Ti (N,O) and SS/Cr-N/Cr (N,O) layers proving the presence of Ti, Cr, O, and N, etc was already reported in our previous investigations [49,50]. Alternatively, the EDS analysis of C-N-TiO_2_-coated SS meshes was conducted using the Oxford instruments (X-Max) detector and data were integrated by Oxford Aztec software suite. The detection of carbon, nitrogen, and titanium distribution in the C-N-TiO_2_ films, mapping elemental images was learned in diverse areas of the sample surface. Images of the surface morphology for each sample were documented at both 30× and 100× magnifications.

X-ray diffraction method (XRD) was used to determine the phase composition of uncoated SS and (SS/Ti (N,O) and SS/Cr-N/Cr (N,O)) coatings (SmartLab diffractometer, Rigaku, Tokyo, Japan), with CuKα radiation (λ = 0.15405 nm). The measurements were taken from 20° to 80°, at a step size of 0.02°. The phase structure of the C-N-TiO_2_ films was studied using a multipurpose X-ray diffractometer D8-Advance from Bruker operated in a continuous theta-theta (θ-θ) scan in locked coupled mode with Cu-Kα radiation (λ = 0.15405 nm). The sample was mounted in the centre of the sample holder on a glass slide and levelled up to the correct height. The measurements run within a 2θ range of 20° to 80° with a typical step size of 0.034°. A positioned sensitive detector, Lyn-Eye, was used to record diffraction data at a typical speed of 0.5 sec/step, which was equivalent to an effective time of 92 sec/step for a scintillation counter. Data were background subtracted so that the phase analysis is carried out for diffraction pattern with zero background after the selection of a set of possible elements from the periodic table. Phases were identified from the match of the calculated peaks with the measured ones until all phases were identified within the limits of the resolution of the results. The size of C-N-TiO_2_ nano crystals was calculated using the Scherrer Equation (1) [35], and the outcomes are presented in Table 1.

(1) $$d=\frac{K\times \lambda }{B\left(2\theta \right)}\;\times \;\cos\theta$$

where *d* is the nano crystal size; *K* ≈ 0.94 is a dimensionless shape factor; *λ* ≈ 0.15406 nm is the CuK_α_ diffraction wavelength; *B* (2θ) is the line broadening at half the maximum intensity (FWHM), expressed in radians (after subtracting the instrumental line broadening); and θ is the Bragg angle in degrees.

Raman spectroscopy (XploRA from Horiba, Kyoto, Japan) was used to elucidate the chemical binding of the prepared C-N-TiO_2_-coated films by probing on the sample surface with continuous 532 nm laser excitation. The Raman spectra were collected from 50–1000 cm^−1^ using a TE cooled CCD camera (Horiba, Kyoto, Japan) attached to the monochromator of a spectrometer with 600 gr/mm. The spectra were obtained by collecting 10 acquisitions.

### 2.3. Applications

#### 2.3.1. Photo Catalysis Investigation

The photo catalytic activity of the C-N-TiO_2_-coated supports including SS and anticorrosion meshes were determined by the decolouration of orange II sodium dye, under solar and UV light as described in Figure 1. Beforehand, the absorption behaviour of control and coated catalysts in O.II dye was verified by running experiments in the dark.

The C-N-TiO_2_-coated meshes (6 cm long and 2 cm large) were individually immersed in 500 mL of 5 mg/L orange II solution in a 1000 mL round glass vessel and consecutively irradiated with solar and UV light at the applied conditions as shown in Figure 1. The photo catalysis system was ice cooled around the vessel. The solution was sampled every 30 min for 2 h and taken for UV-vis analysis at a fixed wavelength of 485 nm. The absorbance recorded was further used to define the decolouration efficiency of orange II dye at each sampling time according to Equation (2).

Decolouration rate % = (A_o_ − A_t_/A_o_) × 100 (2)

where A_o_ is O.II initial concentration at time t = 0 min, and A_t_ O.II is the concentration at sampling time t (min).

#### 2.3.2. Kinetics Investigation

The degradation behaviour of O.II dye was mathematically investigated using the rate constant and half-life kinetic studies according to the following equations:

(3) $$d\frac{\left[O.\mathrm{II}\right]}{\mathrm{dt}}=\;-\mathrm{Kr}\left[O.\mathrm{II}\right]$$

where [O.II] is the concentration of O.II and Kr the rate constant (per minutes).

So Equation (3) was reorganised to Equation (4) as follows:

(4) $$d\frac{\left[O.\mathrm{II}\right]}{\left[O.\mathrm{II}\right]}=\;-\mathrm{Kr}\;\mathrm{dt}$$

At t = 0 min, the concentration of orange II corresponded to [O.II]_0_ and [O.II]_t_ the concentration of orange II at time t. The integration of Equation (4) resulted in the following:

(5) $$\int_{\left[O.\mathrm{II}\right]o}^{\left[O.\mathrm{II}\right]t}\frac{d\;\left[O.\mathrm{II}\right]}{\left[O.\mathrm{II}\right]}\;=\;-\mathrm{Kr}\int_{0}^{t}\mathrm{dt}$$

(6) $$\ln({\left[O.\mathrm{II}\right]}_{o}^{t})=-\mathrm{Krt}$$

(7) $$\Leftrightarrow \ln\left({\left[O.\mathrm{II}\right]}_{t}-{\left[O.\mathrm{II}\right]}_{o}\right)=\;-\mathrm{Krt}$$

(8) $$(\ln\;(\frac{\left[O.\mathrm{II}\right]t}{\left[O.\mathrm{II}\right]o})\;=\;-\mathrm{Krt}\;\Leftrightarrow \;-(\ln\;(\frac{\left[O.\mathrm{II}\right]t}{\left[O.\mathrm{II}\right]o})\;{=}_{\;}\mathrm{Kr}\;t$$

(9) $$\mathrm{Kr}\;t_{1/2}\;=\;-\ln\left(\frac{1/2\left[O.\mathrm{II}\right]o}{\left[O.\mathrm{II}\right]o}\right)\;=\;-\ln\left(\frac{1}{2}\right)\;=\;\ln2\;=\;0.693$$

Therefore, the half-life is

(10) $$t_{1/2}\;=\frac{\ln2}{\mathrm{Kr}\;}=\;\frac{0.693}{\mathrm{Kr}}$$

#### 2.3.3. Antimicrobial Activity

To determine the antibacterial properties of the coatings, the duplicates of the catalyst-coated SS, SS/C-N-TiO_2_, SS/Ti(N,O)/C-N-TiO_2_ and SS/Cr-N/Cr(N,O)/C-N-TiO_2_ (size = 1cm × 1cm) were placed in separate wells of a 24 multi-well plate (Corning, New York city, USA). Uncoated stainless steel (SS) was used as a control. Each well was filled with 3 mL of freshly prepared bacterial culture of *Bacillus subtilis* (SQUMSF005). The initial concentration of the bacterial culture was ~10 Colony Forming Unit per milliliter (~10 CFU/mL). Two similar sets of experiments were conducted; one of which was exposed to the visible light (~30–31.5 Klux, light experiment) and another one was covered with aluminium foil (dark experiment). In both experiments, the multi-well plates were incubated at 37 °C for 48 h. At the beginning (0 h), after 24 h and at the end of the experiment (48 h), 1 mL of the broth culture from each well (both under light and dark conditions) was collected and diluted for 50 times with sterile marine water to determine the number of CFUs. The experiment was conducted in triplicate (*n* = 3).

#### 2.3.4. Statistical Analysis

The Analysis of Variance (ANOVA) followed by the Tukey post-hoc HSD test was used to test the effect of treatment on the number of CFUs of *B. subtilis*. Prior to analysis, Shapiro–Wilk’s test was used to verify the normality of the data. In all cases, a significance level was *p* = 0.05. The calculations were performed using Statistica software version 11.0 (Stat Soft, Austin, TX, USA).

## 3. Results

### 3.1. Scanning Electron Microscopy/Energy Dispersive Spectroscopy

Scanning electron microscopy coupled with electron dispersive spectroscopy (SEM-EDS) analysis was used in order to understand the morphological behaviour/patterns of C-N-TiO_2_ nano-catalyst on SS and the anticorrosion coated metal supports.

**Figure 2:**
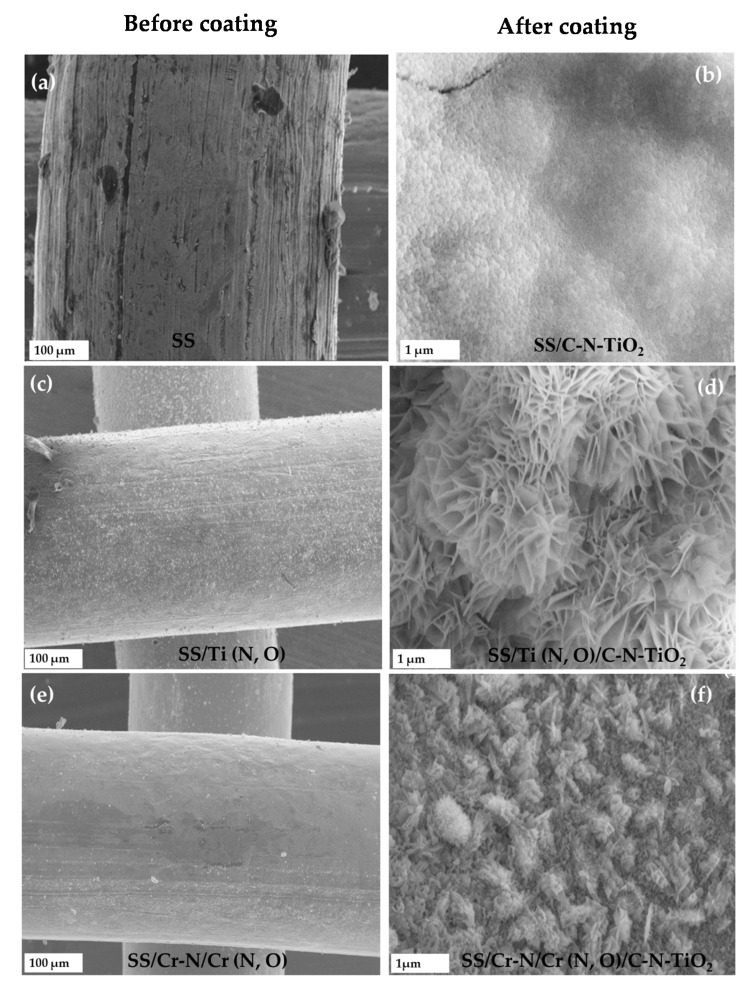
Scanning electron microscopy (SEM) of uncoated (**a**,**c**,**e**) and C-N-TiO_2_ coated (**b**,**d**,**f**) stainless steel and anticorrosion meshes.

The SEM micrographs of the films are presented in Figure 2. The selected rough surface of uncoated SS is shown in Figure 2a, whereas a region coated by C-N-TiO_2_ nano composites is shown in Figure 2b. The SEM micrograph in Figure 2b shows that the morphology of C-N-TiO_2_ appeared in condensed shape on SS substrate. The photo catalytic coating was confirmed by the presence of Ti and N in SS/C-N-TiO_2_ mesh shown by the elemental composition in Table 1. From SEM and EDS results obtained on SS/C-N-TiO_2_ sample, the sol-gel based coating of C-N-TiO_2_ was adhering on SS support and is comparable to the highlights of Passalía et al. [53].

Besides SS, anticorrosion meshes mainly SS/Ti (N,O) and SS/Cr-N/Cr (N,O) coated similarly with C-N-TiO_2_ nano catalyst were used as photo catalytic supports. The SEM-EDS analysis was carried out to investigate the distribution of the nano photo catalyst layer on the selected supports. The SEM characterisation outcomes of SS/ Ti (N,O)/ C-N-TiO_2_ and SS/Cr-N/Cr(N,O) /C-N-TiO_2_ are presented in Figure 2c–f. The SEM micrographs in Figure 2c,d show that C-N-TiO_2_ adhered well to the SS/Ti (N,O) support and appeared as well dispersed nanocrystal. This shows that C-N-TiO_2_ catalyst was stable on SS/Ti (N,O).

As for SS/Cr-N/Cr(N,O)/C-N-TiO_2_, the SEM micrographs in Figure 2e,f show that C-N-TiO_2_ adhered well to the anticorrosion supports. The C-N-TiO_2_ nano crystals that formed on SS/Cr-N/Cr(N,O) support surface exhibited a fine nano rod shape closer to the outcomes reported by Vijayalakshmi and Rajeswari [54].

The EDS results of uncoated discs and those of C-N-TiO_2_ deposited on anticorrosion meshes by pyrolysis of the sol gel layer disclosed are shown in Table 1a,b. Nevertheless, the aim of the EDS analysis before and after immobilization of meshes with C-N-TiO_2_ catalysts was to prove the presence of key elements such as Ti, Cr, O, C, and N in the prepared films. The data in Table 1a indicate that elements Cr, Ti, N, and O were detected in SS and SS/Cr-N/Cr (N,O) and SS/Ti(N,O) anticorrosion coatings, recalling that the corrosion resistance of these coatings has successfully been studied [49,50]. On the other hand, after dip coating immobilisation of C-N-TiO_2_ on the aforementioned supports, the EDS data in Table 1b demonstrate that principal elements including Cr, Ti, O, C, and N were identified. This signified that C-N-TiO_2_ was effectively polished on SS and anticorrosion coatings. The differences in atomic percentages of the elements in Table 1a,b could be attributed to the use of different EDS analytical equipment.

**Figure 3:**
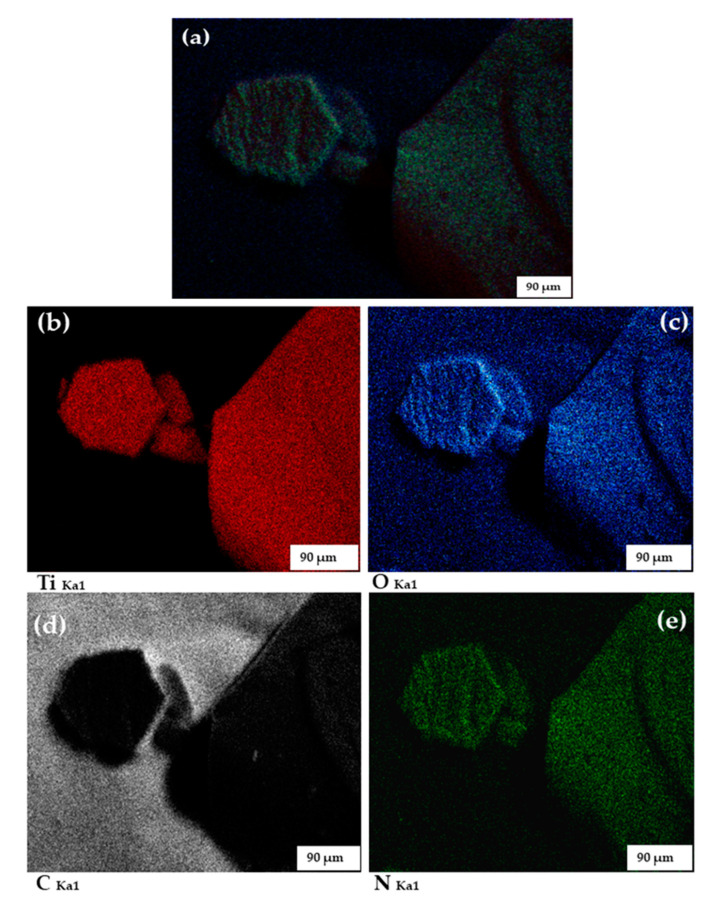
Energy dispersive spectroscopy (EDS) mapping micrographs of SS and anticorrosion meshes coated with C-N-TiO_2_ nano catalyst before photo catalysis process ((**a**) = selected C-N-TiO_2_ coated sample; (**b**–**e**) denote the presence of, Ti, O, C & N, respectively in the C-N-TiO_2_ coated sample).

To provide a visual aid of the distribution of the C and N atoms in the TiO_2_ matrix at the SEM-EDS percentages in Table 1, we conducted the energy dispersive spectroscopy (EDS) mapping of C-N-TiO_2_ nano catalyst on the prepared SS/C-N-TiO_2_ film, and the EDS micrographs are shown in Figure 3. The micrographs presented in Figure 3a–e show that the elements C, N, Ti, and O were all present in the fabricated C-N-TiO_2_ film at percentages dictated by SEM-EDS analysis shown in Table 1b. The morphological changes observed in Figure 2 could be due to the temperature of 350 °C that falls within the temperature range 300 to 400 °C investigated by Tijani et al. [45], which impacted the physical and chemical properties of the synthesised C-TiO_2_ nano composites. This was sustained by Pang et al. [55] who showed that a temperature ranges from 300 to 900 °C had an influence on nano tubes of TiO_2_ morphologies. In summary, during the characterisation process, the SEM images of the coated supports showed that C-N-TiO_2_ photo catalyst was well deposited on SS and the anticorrosion supports. The morphologies of the nano C-N-TiO_2_ on the anticorrosion substrates differed from one another and SS probably only because of the different elemental compositions of each anticorrosion coating and the thermal coefficient of different layers. Hence, the properties of these photo catalytic coatings could also be evaluated.

### 3.2. X-ray Diffraction Analysis of the C-N-TiO_2_ Coated Catalysts

**Figure 4:**
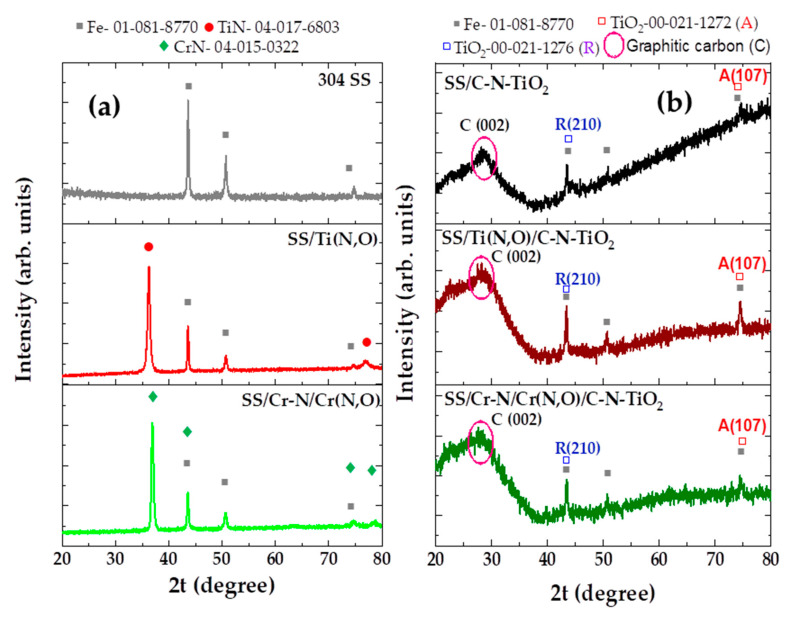
X-ray diffraction (XRD) patterns of: (**a**) 304 SS, SS coated with Ti(N,O) and Cr-N/Cr(N,O); (**b**) C-N-TiO_2_ catalysts coated on SS, SS/Ti(N,O) and SS/Cr-N/Cr(N,O) (pyrolysed at 350 °C, ramping rate 50 °C/min, holding times of 105 min); SS = stainless steel, A = anatase and R = rutile.

The XRD analysis was used to define the phase composition and particle size of SS/C-N-TiO_2_, SS/Ti(N,O)/C-N-TiO_2_ and SS/Cr-N/Cr(N,O)/C-N-TiO_2_ films, and the results are shown in Figure 4. The XRD patterns in Figure 4a show that stainless steel (support) has a major peak with high intensity around 44.05°, a mid-strength peak at 50.65° and a minor peak at 74.03° that could be assigned to Chromium Iron Nickel often referred to as 304-stainless steel (SS) grade. These peaks appear on the SS/Ti (N,O) and on SS/Cr-N/Cr (N,O) coatings as well (Figure 4a), thus it is the support showing through. However, in both SS/Ti(N,O) and SS/Cr-N/Cr(N,O) samples a new intense peak appears at about 36° and a small one at about 75°, characteristics of Ti(N,O) and Cr-N/Cr(N,O) anticorrosion layers previously demonstrated by Dinu et al. [49] and Pana et al. [50].

**Figure 5:**
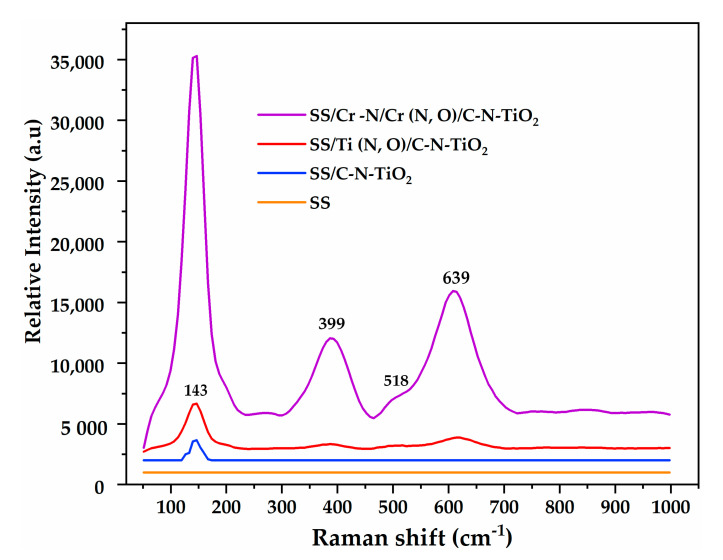
Raman analysis of C-N-TiO_2_ coated films pyrolysed at 350 °C, a ramping rate of 50 °C/min for 105 min.

In Figure 4b, the SS support-related peaks at 44.05°, 50.65° and 74.03° also appear on SS/Ti (N,O)/C-N-TiO_2_, SS/Cr-N/Cr (N,O)/C-N-TiO_2_ and SS/C-N-TiO_2_ coatings. In addition, it can be evidenced that on SS/Ti(N,O)/C-N-TiO_2_, SS/Cr-N/Cr(N,O)/C-N-TiO_2_ and SS/C-N-TiO_2_ difractograms, there is an amorphous hump at around 28° from the carbon in C-N-TiO_2_ nano catalysts that is broad and quite intense, which is typical for graphitic carbon [56,57,58]. The graphitic carbon is more structured in the case of SS/C-N-TiO_2_ than for SS/Ti (N,O)/C-NTiO_2_ and SS/Cr-N/Cr (N,O)/C-N-TiO_2_, and hence, it could be more graphitic. This strongly compliments the Raman spectroscopy results plotted in Figure 5. Indeed, the predominant Raman peaks at 143, 399 and 639 cm^−1^ in Figure 5 identified for SS/Cr-N/Cr(N,O)/C-N-TiO_2_ coating were less featured in SS/Ti(N,O)/C-N-TiO_2_ sample and almost invisible in SS/C-N-TiO_2_ coating except for the peak persisting at 143 cm^−1^, which became minimal and less intense. This trend is further consistent with the EDS outcomes earlier disclosed in Table 1b, showing a significant amount of carbon 8.5 (at.%) in SS/C-N-TiO_2_ compared to 1.7 and 1.6 (at.%) depicted in SS/Ti(N,O)/C-N-TiO_2_ and SS/Cr-N/Cr(N,O)/C-N-TiO_2_ coatings, respectively.

Furthermore, the Rutile and Anatase phases of C-N-TiO_2_ expected to appear at 44.05° and 74.03° might have been hidden by the prominent graphitic carbon in C-N-TiO_2_ nanocomposites [59]. Nevertheless, we believe that the overlapping of rutile and anatase peaks with SS at 2θ = 44.05° (210) and 74.03° (107) and their shadowing by graphitic carbon were responsible for the minimal and broadening of peak intensities as shown in Figure 4b. From this point of view, the outcomes in Figure 4 indicate that C-N-TiO_2_ in the films contained both anatase (JCPDS, no 00-021-1272) and rutile phase (JCPDS, no 00-021-1276). The Rutile phase designated by a diffraction peak at 2θ = 44.05° (210), matched the lattice tetragonal shape of the rutile phase. While the anatase phase occurring at diffraction peak 2θ = 74.03° (107), as from JCPS, no 00-021-1272, is equivalent to the body-centred tetragonal lattice structure of the mineral anatase phase. In order to elucidate the emergence of phase composition of C-N-TiO_2_ nano films, we decided to subject the samples to Raman spectroscopy analysis.

The XRD analysis in Figure 4 depicted not only peaks from the SS support, Ti(N,O) and Cr-N/Cr(N,O) anticorrosion layers, but also the phases of C-N-TiO_2_ layer deposited on SS, SS/Ti(N,O) and SS/Cr-N/Cr(N,O) coatings.

These results meant that C-N-TiO_2_ films fabricated by sol-gel/pyrolysis route were present in both rutile and anatase phases, which may impact the photo catalytic applications of the films owing to the large crystalline size 146 nm, based on the Scherrer equation and enlarged band gap [11,34] compared to 5 nm crystals in anatase phase that were hidden by the graphitic carbon in the synthesised C-N-TiO_2_ nano catalysts.

### 3.3. Raman Spectroscopy Characterisation of the Nano C-N-TiO_2_ Films

The clarification of chemical binding of C-N-TiO_2_ nano films was further investigated by Raman spectroscopy analysis, and the Raman vibrational modes recorded between 20 and 1000 cm^−1^ are shown in Figure 5.

The Raman spectra of irradiated C-N-TiO_2_ nanocomposites in Figure 5 exhibit three major peaks discernable at 143 cm^−1^, 399 cm^−1^ and 639 cm^−1^ and minimal/negligible bands around 198 cm^−1^ and 520 cm^−1^, respectively, which are all characteristics of vibration modes of TiO_2_ anatase phase [60]. The most dominant peak at 143 cm^−1^ is probably due to the symmetric stretching vibration of oxygen atoms in O-Ti-O structure, symmetric bending vibration of O-Ti-O and anti-symmetric bending vibration of *¬*O-Ti-O assembly in *C*-N-TiO_2_ nano catalyst as previously reported [61,62]. The vibrational frequency at 520 cm^−1^ could not be properly examined due to its poor intensity, nevertheless, its shoulder mode could result from the overlapping of two vibration bands that could be depicted at 512 cm^−1^ and 523 cm^−1^, both indicating the anatase phase of TiO_2_ material [63]. For all samples, the outcomes in Figure 5 show that the intensity of vibration peaks declined with the upsurge of Raman frequency, suggesting the progressive reduction of the film’s crystallinity.

Likewise, the intensity of Raman modes captured in irradiated SS/Cr-N/Cr(N,O)/C-N-TiO_2_ nano film gradually decreased in the case of SS/Ti(N,O)/C-N-TiO_2_, SS/C-N-TiO_2_ to a flat surface on SS support. This possibly submits that the amorphisation of carbon in C-N-TiO_2_ was pertinent with SS/C-N-TiO_2_ coating followed by SS/Ti(N,O)/C-N-TiO_2_ and SS/Cr-N/Cr(N, O/C-N-TiO_2_, correspondingly. The redundancy of amorphous carbon in this sample order probably shadowed the occurrence of anatase and Rutile phases of C-N-TiO_2_, which were barely detectable by XRD analysis in Figure 4b. This aspect was attested by a few authors [64,65] who conveyed that the Raman spectrum of amorphous TiO_2_ exhibits no preponderant peaks.

On the other hand, the broad frequency bands noticeable between 720 and 1000 cm^−1^ in SS/Cr-N/Cr(N,O)/C-N-TiO_2_ coating (Figure 5) could perhaps be ascribed to the rutile phase [66], which was previously identified in our XRD analysis though the Raman spectra in Figure 5, showing their total disappearance for SS/Ti(N,O)/C-N-TiO_2_ and SS/C-N-TiO_2_ films. The assignment of these modes between 720 and 1000 cm^−1^ to amorphous phase of TiO_2_ has been previously reported [67,68]. The appearance of these Raman weak and expansive modes in this frequency range suggests the predominance of amorphous edifice over the crystalline one [59]. This corroborates the findings of Hardwick et al. [69], claiming that the shape of Raman spectra can be biased by numerous aspects including phonon confinement, non-stoichiometry due to oxygen deficits, or core strain in the nano-crystallites recalling that phonon captivity influence arises when the estimated grain size of the prepared films lies below 10 nm of those calculated in Table 2. Hence, the diagnostic characterisation of C-N-TiO_2_ films by XRD and Raman spectroscopy reveals that C-N-TiO_2_ on films was predominantly in anatase phase with tiny traces of rutile phase that were shadowed by amorphous carbon and TiO_2_ in C-N-TiO_2_-prepared coatings.

### 3.4. Photo Catalytic Performance of C-N-TiO_2_ Coated SS and Anticorrosion Meshes

**Figure 6:**
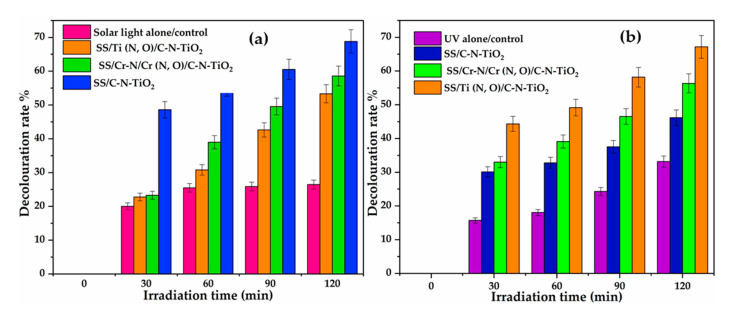
Photocatalytic performance of SS and anticorrosion coatings coated with C-N-TiO_2_ nano composites for the decolouration of orange II (O.II) dye under solar light (**a**) and UV light (**b**). Experimental conditions: Dye concentration 10 mg/L, pH 2.5, volume 500 mL, Mega-Ray 160 W/240 V MR160 UV lamp, and an irradiation time of 120 min (*n* = 3).

The photocatalytic effect was determined by UV-vis analysis of treated water samples drawn from solutions when applying SS, SS/Ti(N,O) and SS/Cr-N/Cr(N,O) meshes coated with C-N-TiO_2_ (SS/C-N-TiO_2_, SS/Ti(N,O)/C-N-TiO_2_ and SS/Cr-N/Cr(N,O)/C-N-TiO_2_) on the % decolouration and rate constant of orange II dye at the applied conditions. The comparative results are presented in Figure 6.

The results in Figure 6a showed that a high percentage of decolouration of orange II dye (69%) was achieved with SS/C-N-TiO_2_ within 120 min of illumination under simulated solar light compared to 58%, 53% and 25% achieved with SS/Cr-N/Cr(N,O)/C-N-TiO_2_, SS/Ti(N,O)/C-N-TiO_2_, and solar light alone, respectively. On the other hand, removal of O.II dye under UV light was still reached with SS/Ti (N,O)/C-N-TiO_2_ (68% in Figure 6b) within 120 min followed by 56, 46 and 32% achieved with SS/Cr-N/Cr(N,O)/C-N-TiO_2_, SS/C-N-TiO_2_, and UV light alone, correspondingly. These results were ascribed to the effectiveness of the C-N-TiO_2_ coatings that achieved over 50% removal of O.II in the allocated time [52]. The photo catalytic efficiency of C-N-TiO_2_ coating demonstrated in Figure 6a is closer to research previously reported [45].

Contrary to our previous investigation [70], we showed that SS, SS/Ti (N,O) and SS/Cr-N/Cr(N,O) anticorrosion coatings are effective catalytic supports. In this study, solar or UV light alone were used as controls. Thus, the results presented in Figure 6 demonstrate that coating SS, SS/Ti(N,O) and SS/Cr-N/Cr(N,O) with C-N-TiO_2_ catalysts improved the removal of O.II about 15-fold more compared to the controls, hence by a ratio of 12:1, which is in accordance with the results reported by Bestetti et al. [71]. These results confirm that layers of C-N-TiO_2_ are photo catalytically effective when deposited on the anticorrosion meshes, which thus can be used as excellent and durable supports. These corroborate the findings reported in previous studies [72,73,74].

### 3.5. Kinetics Trends for the Decolouration of Orange II Dye

**Figure 7:**
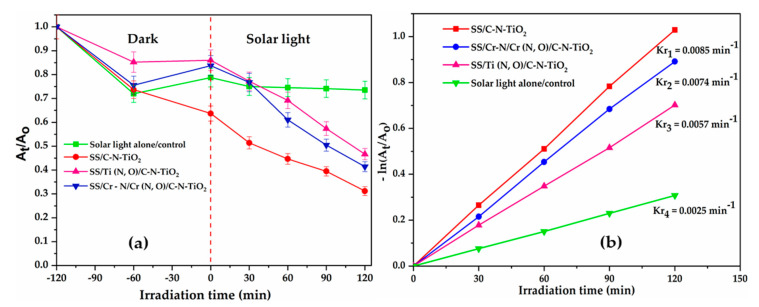

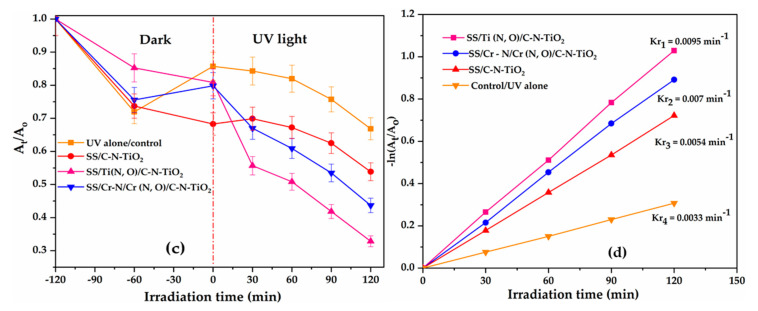
Kinetics trends of the photocatalytic decomposition of O.II dye under solar light (**a**,**b**) and UV light (**c**,**d**) by SS and anticorrosion meshes coated with C-N-TiO_2_ catalysts. Experimental conditions: [O.II] 5 mg/L, pH 2.5, volume 500 mL, Mega-Ray 160 W/240 V MR160 UV lamp, and an irradiation time of 120 min (*n* = 3).

The photo catalysis results previously discussed were complimented by kinetics investigation to further clarify the catalytic effectiveness of the C-N-TiO_2_ coatings. The photocatalytic kinetic results for the decolouration of O.II by SS/C-N-TiO_2_, SS/Ti(N,O)/C-N-TiO_2_, and SS/Cr-N/Cr(N,O)/C-N-TiO_2_ under simulated solar and UV light are plotted in Figure 7a–d.

The kinetic results in Figure 7a,c showed that the photocatalytic decay of orange II dye over time followed a first order reaction rate as expressed in Equations (3)–(8).

In order to assess the kinetic behaviour for the decomposition of O.II over time, −(ln ($\frac{\left[O.\mathrm{II}\right]t}{\left[O.\mathrm{II}\right]o}$) was plotted against time (t) for each photocatalytic system as presented in Figure 7 b,d.

The plot of the first order reaction rate for the decolouration of orange II under solar or UV light is presented in Figure 7b,d and linear trends were observed whose slope corresponded to the rate constant (min^−1^). The rate constant (k_r_) and correlation coefficient (R^2^) of O.II dye decompositions of each oxidation process are presented in Table 3.

Figure 7b shows that the highest rate of removal of orange II under solar light was achieved with SS coated with C-N-TiO_2_ nano composite (SS/C-N-TiO_2_) with a rate constant of 8.5 × 10^−2^ min^−1^ over 120 min of illumination, followed by SS/Cr-N/Cr(N,O)/C-N-TiO_2_, then SS/Ti(N,O)/C-N-TiO_2_ with corresponding rates of 7.4 × 10^−2^ min^−1^ and 5.7 ×10^−2^ min^−1^, respectively.

On the other hand, in Figure 7d, the decolouration rate of O.II under UV irradiation was quicker over SS/Ti(N,O)/C-N-TiO_2_ at a rate of 9.5 × 10^−2^ min^−1^ over 120 min followed by SS/Cr-N/Cr(N,O)/C-N-TiO_2_ and then SS/C-N-TiO_2_ at corresponding rates of 7 × 10^−2^ min^−1^ and 5.4 × 10^−2^ min^−1^, individually. 

In comparison to the previous report on the low or non-existent photocatalytic performance of uncoated support or anticorrosion layers SS, SS/Ti(N,O) and SS/Cr-N/Cr(N,O) [70], the current outcomes substantiated that the immobilised coating of C-N-TiO_2_ on SS support improved the decolouration rate of O.II by 15%, and it is in accordance with Bestetti et al. [71].

Moreover, these results show that the C-N-TiO_2_ coating is photo catalytically effective, and the anticorrosion meshes were suitable to be used as excellent and durable supports, because SS in the absence of anticorrosion layers may corrode in oxidative environments [72,73,74].

These results further endorse that anticorrosion coatings SS/Cr-N/Cr(N,O) and SS/Ti(N,O) can successfully be used as supports for active coatings with the desired catalytic efficiencies in advanced oxidation systems illuminated by UV light or solar light [73]. Indeed, the morphology of C-N-TiO_2_ on SS or on the anticorrosion meshes varied from well dispersed crystals, condensed nano crystals, to nano rod shapes with different surface areas, which all probably absorbed the UV and solar light differently. This consequently implied that the morphology of the catalyst can impact upon its photocatalytic activity [74,75]. Subtle differences in morphology were induced by the underlying anticorrosion coating, which should be further explored. The anticorrosion layers offer a route to prevent SS corrosion in the highly oxidative environment over time [76,77].

In addition to the rate constant discussed in Figure 7b,d, the half-life (t_1/2_) was selected as supplementary indication of the first order chemical decomposition of O.II under solar or UV light. This indicator denotes the time taken for the concentration of orange II to decay to half its initial concentration during the photo catalysis process. Hence, the time taken for concentration of orange II to decline from [O.II]_o_ to 1/2[O.II]_o_ in the first order reaction of each catalyst system was mathematically expressed in Equations (9) and (10).

So, in Equation (8), it could be noticed that from the first order decomposition of O.II, its half-life is independent of its initial concentration. Therefore, at t = 0 min, [O.II] = 10 mg/L decreased to ½ [O.II]_o_ after further integration of ln2/k_r_. Consequently, Equation (9) was used to approximate the half-life of orange II dye during solar or UV illumination in the presence of the composite C-N-TiO_2_ catalysts on SS, SS/Ti(N,O) and SS/Cr-N/Cr(N,O) supports as shown in Table 3.

The decomposition of O.II to half of its concentration took 121 or 72 min with SS/Ti (N,O)/C-N-TiO_2_ coating under simulated solar or UV light. Similarly, it took about 93 or 99 min for O.II concentration to decline to 10/2 = 5 mg/L, when O.II was irradiated under solar or UV light in the presence of the composites SS/Cr-N/Cr(N,O)/C-N-TiO_2_-coated catalyst. Alternatively, about 81 or 128 min corresponded to the time elapsed for O.II concentration to decrease to 2.5 mg/L during solar or UV irradiation with SS/C-N-TiO_2_, respectively.

Furthermore, it would have taken 277 min and 210 min for O.II concentration to go down to 5 mg/L during its irradiation with solar or UV light alone (Figure 7a,c). Thus, the rate constants and half-lives recorded in Table 3 sustain that stainless steel supplemented with its anticorrosion layers could be used as a convenient photocatalytic support in advanced oxidation processes (AOPs), and its subsequent coating with doped heterogeneous nano photo catalysts can significantly improve the removal of persistent organic dye from wastewater, preferably before being discharged into the environment. The nature of the support had an effect on the catalyst due to its impact on the structure of the catalyst.

These results substantiated that the sol-gel/pyrolysis procedure for coating synthesised C-N-TiO_2_ on solid supports such as SS or SS protected with anticorrosion layers could further be utilised as photocatalytic materials under solar light or UV light to enhance the generation of free radicals and hence the removal of the targeted pollutant in AOPs.

### 3.6. Antimicrobial Activity of C-N-TiO_2_ Coated SS and Anticorrosion Meshes

**Figure 8:**
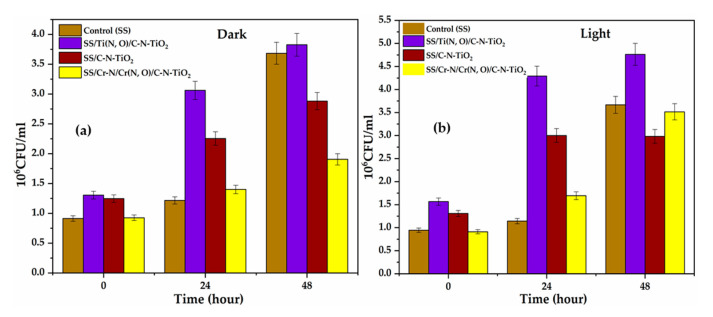
Abundance of *B. subtilis* on SS/C-N-TiO_2_, SS/Ti(N,O)/C-N-TiO_2_ and SS/Cr-N/Cr(N,O)/C-N-TiO_2_ coatings and uncoated stainless steel (control) in dark (**a**) and light (**b**) after 0, 24 and 48 h. Data were means ± SD; *n* = 3.

The antimicrobial activity of stainless steel (SS) (control), as well as the catalyst-coated meshes SS/C-N-TiO_2_, SS/Ti (N,O)/C-N-TiO_2_, and SS/Cr-N/Cr (N,O)/C-N-TiO_2_, were evaluated up on the deactivation of a bacterial culture of *Bacillus subtilis* (*B. subtilis*) in both dark and under visible light at the applied conditions; and the experimental results are shown in Figure 8. The statistical analysis (ANOVA) of the antifouling tests with the C-N-TiO_2_-coated meshes is illustrated in Table 4.

The results in Figure 8 indicate that most of the coatings did not show any significant (ANOVA, Tukey, *p* > 0.05) reduction in the number of viable cells of *B. subtilis* in either light or dark conditions compared to the uncoated stainless steels (control). Nevertheless, the outcomes in Figure 8a show that in the dark, the coating SS/Cr-N/Cr(N,O)/C-N-TiO_2_ followed by SS/C-N-TiO_2_ considerably reduced the number of bacteria after 48 h (ANOVA, Tukey, *p* < 0.0005). This implies that either the bacteria were being absorbed by SS/Cr-N/Cr (N,O)/C-N-TiO_2_ or SS/C-N-TiO_2_ or these two C-N-TiO_2_ coatings were toxic to the bacterium in the dark due to their ability to generate charge carriers (electrons and holes) in the dark that led to substantial decrease of *B. subtilis* colony in the absence of light irradiation.

Conversely, in the light conditions, most of the coatings did not show any reduction (ANOVA, Tukey, *p* > 0.05) in the number of viable cells of *B. subtilis* (Figure 8b) compared to the uncoated SS (control). Yet, various trends could be observed in this case. For instance, there was a continuous growth of bacteria with control/support and SS/Cr-N/Cr (N,O)/C-N-TiO_2_ after 48 h. However, from 0 to 24 h, the number of colony-forming units of *B. subtilis* increased and became constant after 48 h with SS/C-N-TiO_2_. A similar trend was observed with SS/Ti (N,O)/C-N-TiO_2_ though a slight increase in the number of bacteria, which could be observed after 48 h. These probably signified that SS/C-N-TiO_2_ and SS/Ti (N,O)/C-N-TiO_2_ stopped/prevented the growth of bacteria in the presence of light after 24 h onwards. This further implies that SS/C-N-TiO_2_ and SS/Ti (N,O)/C-N-TiO_2_ could biologically be effective catalysts under visible light after extended illumination time. Moreover, with respect to control under visible light after 48 h, it can be observed that SS/C-N-TiO_2_ and SS/Cr-N/Cr (N,O)/C-N-TiO_2_ were slightly more active compared to SS/Ti (N,O)/C-N-TiO_2_ coating. The statistical analysis (ANOVA) displayed in Table 4 suggests that both time and type of coating, as well as their combination, affected the number of viable cells of *B. subtilis*.

Altogether, we found that the three coatings could biologically be effective after extended treatment times, with SS/Cr-N/Cr (N,O)/C-N-TiO_2_ being more toxic to bacteria in the dark while SS/C-N-TiO_2_ responded in both the dark and slightly under visible light. In contrast, SS/Ti (N,O)/C-N-TiO_2_ could be effective under visible light after prolonged exposure time.

Therefore, the C-N-TiO_2_ composites-engineered anticorrosion coatings in this study represent adequate nano thin films that can be utilised in advanced oxidation processes (AOPs) to improve the decontamination of polluted water. These findings have not been reported elsewhere.

## 4. Discussion

The mono and double-layered coatings, SS/Ti(N,O) and SS/Cr-N/Cr(N,O), were identified in our previous investigation as the most corrosion-resistant coatings in acidic environments [49,50]. Thus, the deposition of C-N-TiO_2_ using sol-gel and pyrolysis procedure to form coatings on SS resulted in good adherence of C-N-TiO_2_ coatings at the applied conditions (Figure 2). This can be observed from SEM images shown in Figure 2b, which exhibit the catalyst layer after pyrolysis of the coating, and hence, the coating was present for the photocatalytic irradiation of O.II dye. The absence of Ti in uncoated SS and SS/Cr-N/Cr(N,O) compared to SS/C-N-TiO_2_ and SS/Cr-N/Cr(N,O)/C-N-TiO_2_ coatings or the slight increase of Ti content in SS/Ti(N,O)/C-N-TiO_2_ compared to SS/Ti(N,O), highlighted by the elemental composition in Table 1b, reinforces that C-N-TiO_2_ was successfully immobilised on SS, SS/Ti(N,O) and SS/Cr-N/Cr(N,O) supports. The SEM results, therefore, show that the nanocomposites adhered to supports though extended investigations, proving that positive adherence of the catalyst to the supports over extended usage could be the subject of future studies.

This inferred that the agglomeration of powder nanoparticle issues encountered during water treatment and the problem of particulate recovery after treatment could be overcome by the coating of C-N-TiO_2_ catalysts on supports followed by carbonisation at convenient temperatures and calcination holding times [78,79,80,81,82]. The C-N-TiO_2_ nanocomposites adopted different morphologies for each support. This was related to the physical, chemical and mechanical properties of solid supports SS, SS/Ti(N,O) and SS/Cr-N/Cr(N,O) and their interaction with the nano catalyst [55,77]. Even though the anticorrosion coatings used in this study have the same base/substrate SS, the anticorrosion layers Ti(N,O) and CrN/Cr(N,O) deposited by CAE on SS conferred different properties to the newly fabricated SS/Ti(N,O) and SS/Cr-N/Cr(N,O) coatings when compared to SS substrate [49,50]. So it is evident that deposition of the C-N-TiO_2_ nanocomposites on SS and Ti, Cr nitrides and oxynitride anticorrosion-based layers resulted in various morphologies.

Also, the amorphisation of C and TiO_2_ in C-N-TiO_2_ catalyst, which is demonstrated in Figure 4 and Figure 5, is consistent with SEM images in Figure 2b,d,f in which C-N-TiO_2_ appeared in condensed shape, well-dispersed nano crystals, and fine nano rod shape when immobilised on SS, SS/Ti(N,O) and SS/Cr-N/Cr(N,O) meshes, respectively. This hence confirmed that C and TiO_2_ in C-N-TiO_2_ nano composites were more amorphous on SS than on Ti (N,O) and Cr-N/Cr (N,O) supports, correspondingly. Comparable studies involving the immobilisation of catalysts on supports using different supports and catalysts have been reported [83,84,85,86].

XRD patterns in Figure 4 show that C-N-TiO_2_ coated on SS or anticorrosion coatings was detected as being in rutile phase at 2θ = 44.05° (210), and to some extent the anatase phase was present with the peaks especially depicted at 2θ = 74.03° (107), which is consistent with JCPDS no. 00-021-1276 and JCPDS no. 00-021-1272, respectively. Even though the diffraction peaks identified at 2θ = 44.05°, 50.79°, and 74.03° could also be assigned to SS substrate (JCPDS no. 01-081-8770), the slight increase of peak intensities and their broadening suggest that two phases of C-N-TiO_2_ were present on the supports, and hence, XRD complimented the EDS findings, which are shown in Table 1b and Figure 3. The XRD features of SS have already been discussed in previous studies [87,88], and the illustrated diffraction peaks of SS correspond to the XRD results discussed in Figure 4.

Alternatively, the XRD diffraction peaks observed at 2θ = 44.05° (210) and 74.03° (107) correspond to Rutile and anatase phases. These were previously reported by Cheng et al. [89] and Xie et al. [90] during the synthesis of C-TiO_2_ and N-TiO_2_ catalysts and previous studies support this claim [89,91].

Hence, the large C-N-TiO_2_ nano crystals with a size of 146 nm as shown in Table 2 indicate that all supports were fully covered with the catalyst. It should be noted that small particles/crystals of 5.1 nm size of anatase were barely depicted by XRD analysis, which is certainly due to the predominance of the amorphous C and TiO_2_ in C-N-TiO_2_ nano catalyst identified by the broad and intense XRD hump around 28° and the Raman shift around 143 cm^−1^, 399 cm^−1^ and 639 cm^−1^, consistently. So, the amorphous C and TiO_2_ in C-N-TiO_2_ catalyst coupled with the dominant 146 nm rutile phase may have reduced the catalytic activity of C-N-TiO_2_, resulting in the reduced removal percentages of O.II shown in Figure 6 and slower kinetics trends disclosed in Figure 7. Hence, the SEM, EDS and XRD results discussed in this work show that the immobilisation of catalysts on the tested supports may lead to different crystal morphologies or phases that, in turn, may impact on the photocatalytic activity of the fabricated coatings. Thus, optimisation of the deposition and carbonisation process may be of interest to achieve the desired properties of the films and greater activity.

The photocatalytic outcomes showed that SS, SS/Ti (N,O) and SS/Cr-N/Cr (N,O) were effective supports for active C-N-TiO_2_ coatings after 120 min of photocatalytic illumination. High decolouration rates percentages of 69%, 58%, and 53% of O.II after 120 min time on stream were achieved with SS/C-N-TiO_2_, SS/Cr-N/Cr(N,O)/C-N-TiO_2_ and SS/Ti(N,O)/C-N-TiO_2_, respectively under solar light, as shown in Figure 6a. These findings were complemented by kinetics presented in Figure 7a,b, which show that the fastest O.II removal was reached with SS/C-N-TiO_2_, followed by SS/Cr-N/Cr(N,O)/C-N-TiO_2_, and SS/Ti(N,O)/C-N-TiO_2_ corresponding to first-order rate constants of 8.5 × 10^−3^ min^−1^, 7.4 × 10^−3^ min^−1^, and 5.7 × 10^−3^ min^−1^, respectively. Moreover, the half-life findings indicated that it took 81 min for O.II to decompose to its half concentration with SS/C-N-TiO_2_ catalyst, while 93 and 121 min were required to degrade O.II dye to its half-concentration with SS/Cr-N/Cr(N,O)/C-N-TiO_2_, and SS/Ti(N,O)/C-N-TiO_2_, correspondingly.

Alternatively, the results in Figure 6b show that the best removal of orange II 68%, under UV light was achieved with SS/Ti(N,O)/C-N-TiO_2_ film seconded by 56% reached with SS/Cr-N/Cr (N,O)/C-N-TiO_2_ and 46% attained with SS/C-N-TiO_2,_ corresponding to first order decolouration rates of 9.5 × 10^−2^ min^−1^, 7 × 10^−2^ min^−1^ and 5.4 × 10^−2^ min^−1^, respectively. In this regard, the abatement of O.II dye required 72 min, 99 min, and 128 min to decompose to its half-concentration with SS/Ti(N,O)/C-N-TiO_2_, SS/Cr-N/Cr(N,O)/C-N-TiO_2_, and SS/C-N-TiO_2_, separately.

**Figure 9:**
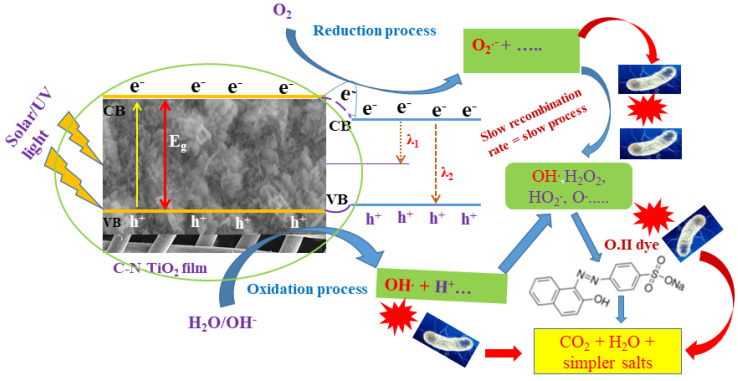
Schematic showing the generation of reactive oxygen species (ROS) during photo catalytic detoxification of O.II dye and inactivation of *B. subtilis bacteria*.

The photocatalytic process occurring during the destruction of O.II under both solar and UV light irradiation was induced by reduction of O_2_ to O_2_**^−^** and OH by excited e**^−^** on the conduction band (CB) on the surface of the catalyst followed by oxidation of water molecules (H_2_O/OH**^−^**) to OH radicals on the Valence band (VB). The resultant active species O_2_**^−^**, OH**^.^**, etc., further attacked and decomposed O.II dye to CO_2_, H_2_O and simpler inorganic entities, as described in Figure 9.

Sambandam et al. [92] reported that the anatase phase is photo catalytically more effective than the rutile phase due to rapid electron-hole recombination and probably lower surface activity of rutile phase.

This consequently led to a reduced number of microstates that in return resulted in quick electron-hole pair recombination (λ_1&2_) as shown in Figure 9. This diminished the storing of e**^−^** on the catalyst surface and hence lowered reduction of O_2_ to O_2_**^−^** and OH. This in turn decelerated the decolouration of O.II dye to percentages below 80%.

Similar studies on sol-gel deposition of TiO_2_-doped or co-doped catalysts on SS have been conducted and high decolouration efficiencies of POPs were also achieved [45,93,94,95,96]. SS has been proven unstable in acidic environments due to the erosion of its passive layer that often leads to its corrosion [45,54]. The new materials also offer a route to prevent SS corrosion in the highly oxidative environment over time [75].

The different photo catalytic activities of C-N-TiO_2_-coated nano films SS/C-N-TiO_2_; Cr-N/Cr (N,O)/C-N-TiO_2_ and SS/Ti (N,O)/C-N-TiO_2_ observed in both solar and UV light, as shown in Figure 6 and discussed above, could be ascribed to the coordination chemistry that involves inorganic semiconductor–insulator, inorganic semiconductor–semiconductor, and inorganic semiconductor–metal interactions with doped semiconductor nanomaterials of C-N-TiO_2_ that were earlier described by Li and Zhang [97]. These chemical interfaces consequently affected the optical and electronic properties of C-N-TiO_2_ films, leading to different activities under solar and UV light illumination. The understanding of these chemical interactions in the current study requires full investigation and will be considered as part of our future studies. Besides the photocatalytic properties of C-N-TiO_2_ coated on SS and anticorrosion meshes discussed above, investigation of antibacterial characteristics of the coating could be crucial to evaluate whether during decomposition of POPs in polluted effluents, the C-N-TiO_2_ coating catalysts might eliminate microbes from effluents being remediated.

Hence, the second test of our coatings involved assessing antimicrobial activities towards the *Bacillus subtilis* in the dark and under visible light. Indeed, Chang et al. [98] and Cai et al. [99] demonstrated that Cr-N anticorrosion layers do not have any antibacterial activity. On the other hand, Li and Zhang [97] claimed that TiO_2_ photo catalysts doped with C, N, S, or F anionic impurities exhibit great photocatalytic efficiencies, but they often lose their photocatalytic ability in the dark milieu because they cannot produce electron and hole-pairs. Nevertheless, the antimicrobial activity/properties/behaviour of SS/Cr-N/Cr (N,O)/C-N-TiO_2_ and SS/C-N-TiO_2_ films experienced in the dark in Figure 8a could likely be attributed to their capability to generate charge carriers (electrons and positive holes) as a consequence of optoelectronic coupling between C, N dopants and TiO_2_ semiconductor, which promotes the charge carrier separation in C-N-TiO_2_ nanocomposites. Previous research studies [100] highlighted that the ideal scenario would require the fabrication of photo catalysts with high activity under visible/UV light and when the photo excitation process is turned off so that contaminants such as bacteria can be cleaned up either in the presence or absence of light.

The photocatalytic mechanistic scenarios plotted in Figure 9 indicate that the irradiation of the co-doped C-N-TiO_2_ nanocomposites with solar/UV light initiated the excitation of electrons from the valence band (VB) to the conduction band (CB), leaving behind positively charged empty holes (h^+^) that both contributed to the production of reactive oxygen species (ROS). Gao et al. [101] and Ajiboye [102] recalled that the indirect inactivation of bacteria such as *B. subtilis* described in Figure 9 is often initiated by the damage of the plasmonic membrane, which may alter the bacteria metabolism, followed by the destruction of DNA sequence leading to its lysis.

Indeed, the electrons stored on the CB of C-N-TiO_2_ film participated in the reduction of O_2_ to superoxide anions (O_2_^−^), which directly attacked the plasmonic membrane of *B. subtilis* leading to its deactivation and hence the slight reduction of its colony-forming unit counts observed in Figure 8b with SS/C-N-TiO_2_ after 48 h. Similar investigations were carried out using different advance oxidations [103,104,105,106,107,108,109,110,111].

The extended inactivation time of *B. subtilis* suggests that OH oxidants originated from various chains of chemical reactions at low rates between O_2_**^−^**, H_2_O_2_ and other species after 2 h and are in line with photo catalysis results discussed in Figure 5 and Figure 6. This implies that in the case of bacteria resistance, prolonged deactivation time is required as observed in Figure 8.

Besides, O_2_^−^ oxidants also contributed to the generation of powerful non-selective OH radicals that are engaged in both decomposition of POP dye O.II and deactivation of *B. subtilis*. The decomposition of O.II in Figure 9 was probably initiated by OH, and the results agree with [112]. On the other hand, the empty hole charge carriers on the VB of C-N-TiO_2_ nanocatalyst oxidised H_2_O molecules to OH**^.^**. These in return participated in both dye decolouration and inactivation of B. subtilis after extended irradiation times. Secondary species including O**^.^** and H_2_O_2_ might have also been involved in reaction chains producing an O_2_^−^ and OH radical during the photo catalysis process and during deactivation of *B. subtilis* [113,114].

In contrast, the mechanisms of action of C-N-TiO_2_-coated films on *B. subtilis* inactivation in visible light shown in Figure 9 may not only result from the ROS generated but also from the photo chemical irradiation of water that further induced water disinfection via sterilisation of *Bacillus* micro-organisms (pathogens). Furthermore, Ruddaraju et al. [115] noted that nanoparticles can modify the metabolic behaviour of bacteria when interacting directly with bacterial cells via electrostatic interaction, van der Waals forces, receptor-ligand, and hydrophobic contacts, which is in line with Choi et al. [116].

This study demonstrated that coating SS mesh with transition metals and non-metals in mono and double protective layers SS/Ti(N,O) and SS/Cr-N/Cr(N,O) contributed not only to the protection of stainless steel against corrosion in the oxidative photolytic environment but also made excellent photocatalytic supports. The morphology of the catalyst being deposited may vary according to its adherence due to the composition of the support, which in turn could be influenced by interfacial issues or thermal properties that could be further investigated.

## 5. Conclusions

This study showed successful coating of C-N-TiO_2_ nano composite layers upon supports, being obtained by pyrolysis of a sol-gel TiCl_4_/PAN/DMF dip coated onto the SS support or onto pre-prepared anticorrosion meshes. The C-N-TiO_2_ coating exhibited different morphologies and the crystal habit varied from well dispersed, condensed-shaped crystals to dispersed nano rods. The C-N-TiO_2_ immobilised on SS or onto anticorrosion meshes was predominantly in the rutile phase with a crystal size of 146 nm compared to a minor phase of anatase present with particle size of 5 nm. The photo catalytic efficiency of C-N-TiO_2_-coated catalysts for the removal of O.II dye was between 70% and 32% under both solar or UV light.

The decolouration of O.II at these percentages followed a first order reaction rate characterised by linear trends. The fabricated C-N-TiO_2_ films showed significant antibacterial activities in both dark and visible light at prolonged treatment times. Herein, we proved that morphology, phase and crystal size of the catalyst immobilised on SS and anticorrosion supports impact on the photocatalytic capabilities of the coatings and were advantageous for the deactivation of microorganisms in the presence or absence of light.

This is the first time that C-N-TiO_2_ nano catalyst synthesised by sol-gel method was immobilised on SS and various anticorrosion meshes that resulted in different morphologies with improved photo catalytic activities. The chemical coordination between the immobilised nanocatalyst and supports might have affected the optical and electronic properties of the films, which in return led to different photo catalytic activities. The C-N-TiO_2_ coatings engineered in this study can be used in water and wastewater treatment plants for the decomposition of POPs under both solar and UV light and for the killing of bacteria in both dark and light.

## Figures and Tables

**Table 1:** a: Normalised elemental composition of coated stainless steel before photo catalysis (at.% = atomic percentage); b: Normalised elemental composition of C-N-TiO_2_ coated stainless steel and anticorrosion meshes after photo catalysis.

a									
Anticorrosion Samples	Elemental Composition (at. %) before Immobilisation of C-N-TiO_2_ Nano Catalyst								
	Ti	Cr	N	O	C	Fe	Mn	Ni	Si
SS/Ti(N,O)	49.7	-	43.0	7.3	-	-	-	-	-
SS/Cr-N/Cr(N,O)	-	49.1	37.2	13.7	-	-	-	-	-
**b**									
Coated Samples	Elemental Composition (at. %) after Immobilisation of C-N-TiO_2_ Nano Catalyst								
	Ti	Cr	N	O	C	Fe	Mn	Ni	Si
SS/C-N-TiO_2_	11.5	6.5	7.0	35.8	8.5	10.3	6.1	8.6	5.8
SS/Ti(N,O)/C-N-TiO_2_	41.5	3.8	6.3	32.6	1.7	13.3	0.3	0.4	0.1
SS/Cr-N/Cr(N,O)/C-N-TiO_2_	33.4	3.2	6.6	31.1	1.6	19.6	0.4	2.6	0.5

**Table 2:** Particle sizes of C-N-TiO_2_ coated on SS, SS/Ti (N,O) and SS/Cr-N/Cr (N,O) anticorrosion meshes calculated from XRD analysis using the Scherrer equation (*n* = 2).

Photo Catalysts.	Catalyst Grain Size (nm)	
	Rutile	Anatase
SS/C-N-TiO_2_	±146	±5.1
SS/Ti(N,O)/C-N-TiO_2_	±146	±5.1
SS/Cr-N/Cr(N,O)/C-N-TiO_2_	±146	±5.1

**Table 3:** Kinetic parameters for the decolouration of orange II dye at the following conditions: [O.II] 5 mg/L, pH 2.5, solution volume 500 mL, Solar simulator light (AM 1.5 radiation, 100 mW/cm^2^) and Mega-Ray 160 W/240 V MR160 UV lamp, and an irradiation time of 120 min.

C-N-TiO_2_ Coated Meshes	Rate Constant (Kr/min)		Correlation Coefficient (R^2^)		Half-Lifetime (min)	
	Solar Light	UV Light	Solar Light	UV Light	Solar Light	UV Light
Solar/UV alone (control)	0.0025	0.0033	0.9914	0.9943	277.20	210
SS/C-N-TiO_2_	0.0085	0.0054	0.9992	0.9969	81.53	128.33
SS/Ti(N,O)/C-N-TiO_2_	0.0057	0.0095	0.9984	0.9971	121.57	72.94
SS/Cr-N/Cr(N,O)/ C-N-TiO_2_	0.0074	0.007	0.9989	0.9994	93.65	99

**Table 4:** ANOVA results for CFU comparisons during the antibacterial bioassay with *B. subtilis* in solar light and dark obtained with SS/C-N-TiO_2_, SS/Ti(N,O)/C-N-TiO_2_ and SS/Cr-N/Cr(N,O)/C-N-TiO_2_ and uncoated stainless steel (control) at varied experimental times 0, 24 and 48 h (SS–sum of squares; DF–Degrees of freedom; MS–mean of squares; F–F-statistic; P–probability).

Antimicrobial Bioassay under Solar Light					
Source	SS	DF	MS	F	P
Time	0.051970	2	0.025985	3057.85	0.000094
Coatings	0.007191	6	0.001198	141.03	0.000003
Time × Coatings	0.011994	12	0.001000	117.62	0.000004
Error	0.000051	6	0.000008		
**Antimicrobial Bioassay in the Dark**					
Source	SS	DF	MS	F	P
Time	0.320476	1	0.320476	22632.51	0.000006
Coatings	0.027186	2	0.013593	959.96	0.000003
Time × Coatings	0.035304	6	0.005884	415.54	0.000001
Error	0.017702	12	0.001475	104.18	0.000006
	0.000085	6	0.000014		
